# Study on the Preparation of Network Ti-N/Ti Composites by Nitridation of Ti Powders

**DOI:** 10.3390/ma16155259

**Published:** 2023-07-26

**Authors:** Ziyang Xiu, Boyu Ju, Junhai Zhan, Weidi Chen, Aiping Yin, Xiaolin Zhu, Pengjun Wang, Ping Wu, Wenshu Yang

**Affiliations:** 1 State Key Laboratory of Advanced Welding and Jointing, Harbin Institute of Technology, Harbin 150001, China; yws001003@163.com; 2 Shanghai Aerospace System Engineering Research Institute, Shanghai 201108, China; jpy_mat@163.com (J.Z.); weidichen_hit@sina.com (W.C.); aipingyin_hit@163.com (A.Y.); xiaolinzhu_hit@sina.com (X.Z.); 3 Xian Honor Device Co., Ltd., Xi’an 710000, China; wangpengjun9@163.com; 4 Key Laboratory of Advanced Science and Technology on High Power Microwave, Xi’an 710024, China; wuping2007ssss@163.com; 5 Northwest Institute of Nuclear Technology, Xi’an 710024, China

**Keywords:** TiN, Ti matrix composite, network composite, mechanical properties

## Abstract

Composite structure design is an important way to improve reinforcement strengthening efficiency. The dispersion of the external reinforcement is often not uniform enough, however, and it is agglomerated in the matrix, which cannot uniformly and effectively bear the load. The interconnected reinforcement network prepared by the in-situ self-growth method is expected to obtain higher material properties. In this paper, the TiN shell was formed on the surface of Ti powder by the in-situ nitriding method, and then the network TiN/Ti composites were prepared by sintering. In the control group, TiN was dispersed by mechanical ball milling, and it was found that TiN powder was coated on the surface of Ti particles, and the sintered TiN/Ti composites formed a discontinuous structure with a great deal of TiN agglomeration. A uniform TiN nitride layer of 5~7 μm was formed on the surface of Ti powder by the in-situ nitriding method, and a connected TiN network was formed in the sintered Ti-N/Ti composites. The composites prepared by nitriding have higher compressive strength, hardness, and plasticity. The hardness of the Ti-N/Ti composite is 685.7 HV and the compressive strength is 1468.5 MPa. On this basis, the influence of the connected TiN structure on the material properties was analyzed, which provided theoretical guidance for the structural design of the network structure-reinforced titanium matrix composites.

## 1. Introduction

In recent decades, with the rapid development of automobiles, aerospace, shipbuilding, medicine, chemicals, and other fields, the performance of materials has also made different degrees of progress on the basis of traditional materials [1,2,3]. Titanium and its alloys have good comprehensive properties. Due to its high strength, low density and environmental friendliness, titanium is widely used in the aerospace, biomedical, and transportation fields [4,5,6]. Titanium matrix composites are a new material system prepared by adding reinforcements to the Ti matrix. The composite design combines the ductility and toughness of titanium and its alloy matrix with the high modulus and high strength of the reinforcing phase to obtain better mechanical properties and better high-temperature physical and chemical properties [7,8].

TiN is a ceramic reinforcement with high chemical stability. The coefficient of thermal expansion (CTE) of TiN is close to that of the titanium matrix and has a high elastic modulus. It is expected to improve the properties of titanium matrix composites [9]. Ti-based composites reinforced by TiN and TiC were studied by Xi et al. [10] It was found that the properties of the reinforcement were well-developed due to the good interface bonding between the reinforcement and the matrix, and the hardness of the composites was greatly increased compared with the matrix. Li et al. [11] prepared nano-TiN reinforced Ti matrix composites by the powder metallurgy method, using an N_2_ and Ti in-situ reaction to form 20–100 nm TiN, so that the hardness increased by 101% compared with the matrix. Cui et al. [12] prepared a TiN/Ti coating on the Ti surface by laser. It found that a good metallurgical bonding was formed between the TiN reinforced phase and titanium substrate, and the hardness and wear resistance were improved. In the above study, the morphology of TiN was granular and dispersed in the matrix.

The new research found that when the reinforcement forms a connected structure, it can transfer the load more effectively, so that the performance of the composite material has a new breakthrough [13]. Zhang et al. [14] prepared three-dimensional connected graphene/Cu composites. It is found that the three-dimensional connected network structure can effectively transfer load, improve the mechanical properties of materials, and contribute greatly to electron conduction. Chu et al. [15] prepared high-content graphene/Cu composites (graphene content exceeded 35 vol.%). It was found that at a high volume fraction, graphene formed a connected network in the material, which increased the thermal conductivity of the composite by 50% compared with the Cu matrix. Ozerov et al. [16] used the reaction of TiB_2_ with Ti at a high temperature to generate short TiB whiskers in situ in composites. The generation of TiB results in a substantial increase in the hardness and frictional wear properties of the composite. 

Wang et al. [17] used a rapid sintering process to prepare reticular graphene nanosheets and TiB whisker reinforced titanium matrix composites. By designing the network structure of graphene and TiB, the interface bonding was improved, and the preparation of high-plasticity titanium matrix composites was achieved. The tensile strength reached 1158 MPa, and the plasticity reached 15.8%, which is consistent with the plasticity of the matrix alloy. Zhang et al. [18] prepared (La_2_O_3_ + TiB)/Ti_2_AlNb composites using in-situ borides. Prior particle boundaries were interconnected to form a connected network structure to avoid premature cracking of the material in the elastic stage, which was conducive to the improvement of the overall strength and plasticity. Li [19] and Wang et al. [20,21,22] prepared network TiB/titanium matrix composites in different titanium-based alloys. They all found that this network structure is beneficial to the grain refinement of the matrix and the load transfer of TiB, which has a positive contribution to the material properties. In-situ autogenous TiN and Ti_2_N by nitriding can also provide reinforcement in composites. Traxel et al. [23] reacted Ti with BN to generate TiN and TiB in situ autogenously, which increased the yield strength from 480 MPa to 610 MPa. Candela et al. [24] used direct laser deposition to self-generate TiN in situ on Ti, which increased the microhardness of the composite by 50–100%. In-situ autogenous TiN on Ti surface was mainly applied in the direction of coating surface research [25,26,27] to substantially improve surface hardness, friction, and wear properties, and possess good bonding ability. Sahasrabudhe et al. [28] found that nitrides grow on the surface as dendrites during the nitriding of Ti surfaces; composites with TiN and Ti_2_N showed higher hardness and wear resistance compared to the untreated Ti substrate. 

It can be seen that the connect structure has many applications in the design of composites, and the connected structure has unique advantages in load transfer and electrical heat conduction [14,29,30]. Similarly, TiN coating on the surface of Ti powder to form a network structure is also expected to obtain higher material properties. However, the research on the process of preparing three-dimensional connected TiN reinforced Ti matrix composites by the in-situ method is not deep enough, and the contribution of connected structure to the mechanical properties of materials is not clear. For this reason, we prepared composites using SPS to study the strengthening effect of reticulated TiN. Spark Plasma Sintering (SPS) is a new technology that uses pulse current to achieve rapid sintering of powder. Due to the short sintering time and no need for a long time holding, the composites prepared by SPS often have less interfacial reaction and higher sintering efficiency. The material has high density and mechanical properties [31,32].

In this paper, Ti-N/Ti composites with network structure were prepared by in-situ nitriding. TiN/Ti prepared by simple mechanical ball milling was used as a control. The microstructure evolution process of materials under different processes was studied, and the compressive properties of composites were tested. The special effect of core-shell structure on material properties is also discussed.

## 2. Materials and Methods

### 2.1. Raw Materials

**Figure 1:**
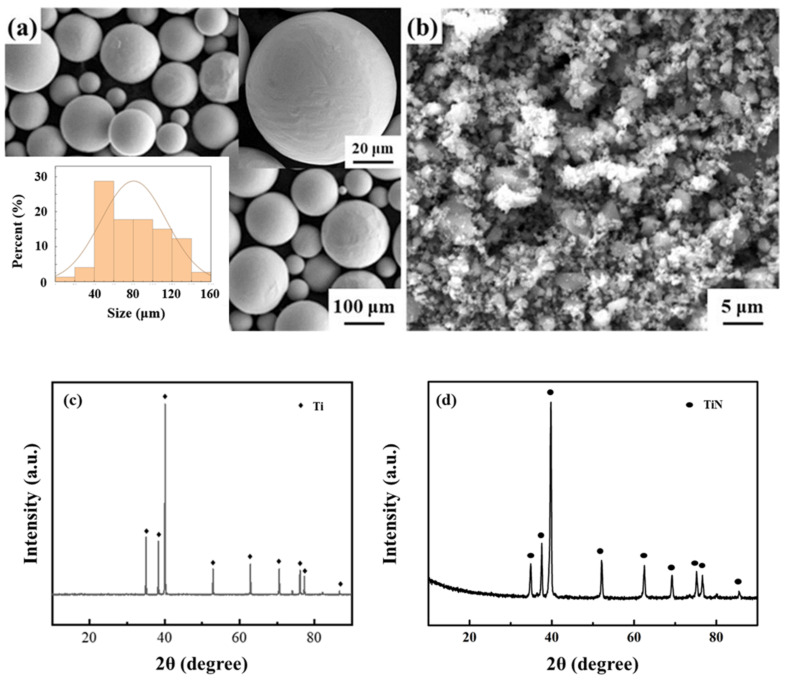
Morphology and XRD characterization of raw powders. (**a**,**c**) Ti powder; (**b**,**d**) TiN powder.

The Ti powder studied was spherical, with an average diameter of about 80 μm, as shown in Figure 1a. The TiN used in the control group was an irregular polygon with an average diameter of 3 μm. The diameter of TiN was much smaller than that of Ti powder, which ensured that TiN could be uniformly wrapped on the surface of Ti powder during ball milling to achieve effective dispersion. The XRD characterization results are shown in Figure 1c,d.

### 2.2. Preparation of Ti-Based Composites

**Figure 2:**
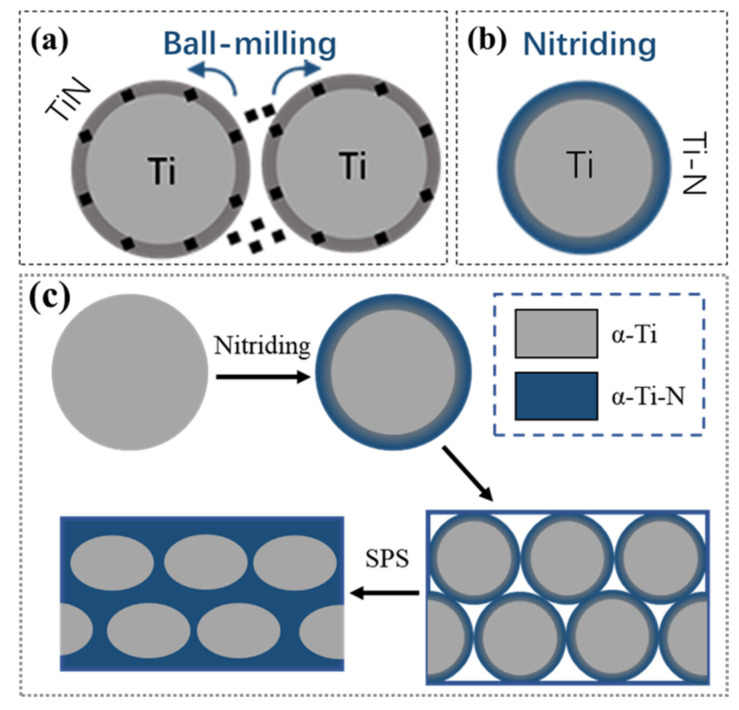
Schematic diagrams for the preparing of mixed powders with core-shell microstructure. (**a**) TiN/Ti mixed powders prepared by ball milling; (**b**) Ti-N/Ti mixed powders fabricated by nitriding; (**c**) the process diagram of the preparation of network Ti-N/Ti composites by SPS sintering.

In this paper, the effect of the distribution mode of TiN on the material properties is thoroughly investigated. Ti-N powders with core-shell structures were prepared by the Ti powder nitriding process, which in turn led to the preparation of Ti-N/Ti composites with a reticular connectivity structure. The control group used the mechanical ball milling process to prepare non-connected TiN/Ti composites. Two approaches were used to obtain the Ti/reinforcement mixed powder, as shown in Figure 2. The organizational changes, strengthening effects, and performance advantages of network structures compared to non-connected structures are investigated.

**Figure 3:**
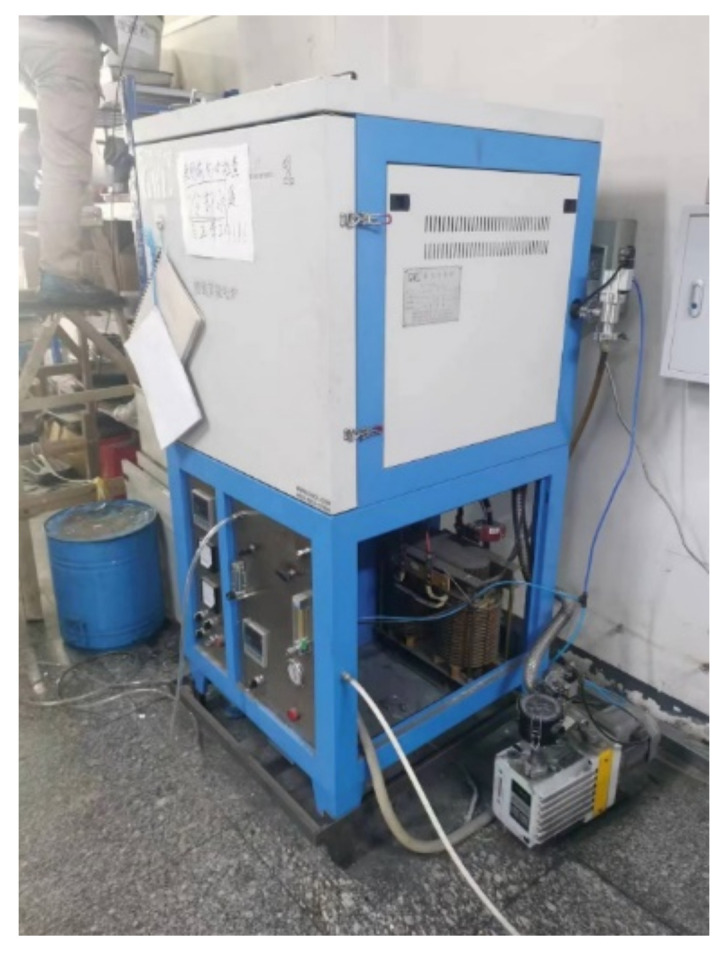
Picture of a self-made nitriding furnace. On the basis of the vacuum sealing furnace, the vacuum pump and nitrogen gas cylinder device are connected.

The Ti powder and TiN powder used in the experiments were provided by the Northwest Institute for Nonferrous Metal Research, Xi’an, China. This experiment investigated the effect of the TiN distribution mode on the properties. Among them, Ti-N powders with core-shell structures were prepared by nitriding Ti powders. The preparation process involves laying the Ti powder uniformly and loosely flat in an Al_2_O_3_ high-temperature ceramic container and placing it in a furnace for high-temperature nitriding. For the nitriding process, the nitriding temperature and time were 880 °C and 480 min, respectively. The chamber of the nitriding furnace was repeatedly cleaned three times before nitriding to avoid the influence of residual air. The purity of nitrogen used in the nitriding process was 99.99%, and the flow rate was 200 mL/min. Afterwards, the Ti-N/Ti mixed powder was prepared by the nitriding of the pure Ti powder in the self-made nitriding furnace (as shown in Figure 3), as sketched in Figure 2b. The content of Ti-N compounds in the mixed powder was obtained by quantitative metallography.

The control group was TiN/Ti composites prepared by a mechanical ball milling process. For TiN/Ti mixed powder, the additive amount of reinforcement was 30 vol%. The TiN coating on the surface of the Ti powder was achieved by mechanical ball milling, as shown in Figure 2a. The equipment used was planetary ball mill apparatus QM-3SP2, Instrument Factory of Nanjing University, China, Nanjing. The rotation speed, milling time, milling atmosphere, and ball-to-material ratio were 290 r/min, 8 h, argon gas, and 5:1, respectively. 

The powders prepared by ball milling and nitriding were sintered into materials by SPS. The powder was loaded into a high-density graphite mold and sintered under vacuum conditions. The SPS equipment model was FCT HPD-250, Germany. The sintering temperature and holding time were selected as 1200 °C and 30 min, respectively. The sintering process maintained a pressure of 40 MPa to increase the density of the material. The vacuum degree was less than 8 Pa, and the pores in the auxiliary material were discharged.

### 2.3. Microstructure Characterization of Ti-Based Composites

The phase composition of the composites was characterized by X-ray diffraction (XRD). The XRD equipment used was the Empyrean Intelligent X-Ray Diffractometer (Spectris Co., Ltd., Shanghai, China). During the test, the voltage was 40 kV, the current was 40 mA, the target was Cu-Kα, and the scanning speed was 10°/min to ensure that the results had high accuracy.

The microstructure of powders and composites was observed by optical microscope (OM) and scanning electron microscope (SEM). When the powder was tested, it was uniformly dispersed on the conductive adhesive for SEM observation. The composite material was cut into 10 mm × 10 mm × 2 mm sheets, and the surface was polished and cleaned for OM and SEM observation. The equipment used was the ZEISS metallographic microscope and the Quanta 200FEG field-emission scanning electron microscope (Thermo Fisher Scientific Co., Ltd., Waltham, MA, USA). During the SEM observation, the energy-dispersive spectrometer (EDS) was used to assist the composite material to characterize the composition, and the structural analysis was carried out in combination with the XRD results. 

### 2.4. Performance Tests of Ti-Based Composites

The compressive properties of the composites were tested afterwards. Compression tests were performed using the Instron-8862 universal electronic testing machine (Instron, Norwood, MA, USA). The compression rate was 0.25 mm/min. Each sample was tested three times to ensure high reliability of the results.

Hardness was tested by means of a Vickers Hardness Tester HV-200 (Vickers, London, UK). The surface of the sample was polished with 2000# sandpaper and then five random hardness points were tested and averaged. The test load was 300 N and the holding pressure was 15 s.

A cylinder with a size of Φ12.7 mm × 3.2 mm was processed by wire cutting, and the upper and lower surfaces of the specimens were polished with 400#, 800#, and 1000# water sandpaper to ensure that the surfaces were smooth and even, and the upper and lower surfaces of the polished surfaces were uniformly coated with toner to ensure that the surfaces of the specimens were uniformly heated during the test. The thermal conductivity test was carried out on the LFA 447 NanoflashTM laser thermal conductivity meter produced by NETZSCH Company in Selb, Germany, which tested the thermal diffusion coefficient of the composite material samples at room temperature (25 °C) α. The law of mixtures was utilized to calculate the specific heat capacity of the composite material, C. The thermal conductivity of the composite was determined by the product of the density of the composite, the specific heat capacity, and the thermal diffusion coefficient. The calculation method was as follows:

λ=k×ρ×C

*λ*—thermal conductivity, W/(m·K),
*k*—thermal diffusion coefficient, m^2^/s,
*ρ*—density of composite, kg/m^3^,
*C*—specific heat capacity, J/(kg·K).

## 3. Result and Discussion

### 3.1. Microstructure Characterization of TiN and Ti Mixed Powders Prepared by Ball Milling and In-Situ Nitriding

**Figure 4:**
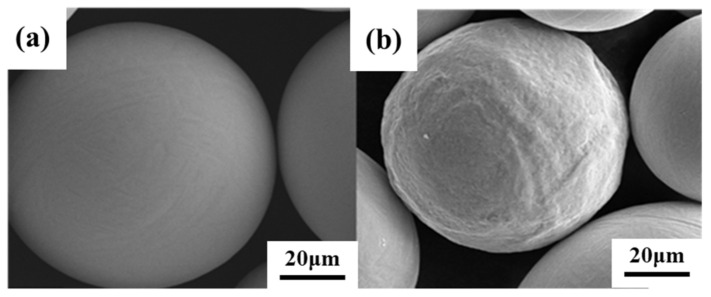
SEM characterization of Ti powder. (**a**) Original Ti powder; (**b**) nitrided Ti powder.

**Figure 5:**
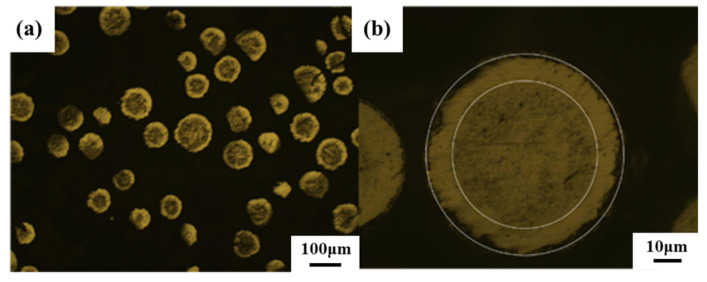
Metallographic microscope observation of nitrided Ti powder. (**b**) is an amplification of (**a**).

**Figure 6:**
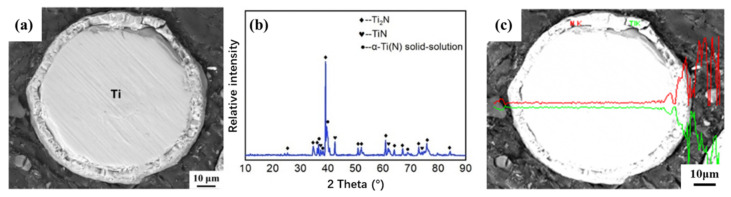
Cross-sectional morphology of nitrided Ti powder and corresponding XRD patterns and EDS characterization. (**a**) SEM characterization of Ti powders with core-shell structure. (**b**) TEM characterization. (**c**) Line-scan EDS characterization of the cross-section of Ti powders with a core-shell structure. The abundances of N and Ti are represented by red and green lines, respectively.

The morphology of nitrided Ti powder is shown in Figure 4, Figure 5 and Figure 6. From the SEM characterization, it can be seen that the surface smoothness of Ti powder decreased significantly after nitriding, and a coating layer was grown. Under the metallographic microscope, it can be seen that the Ti powder exhibits a core-shell structure characterized by a shell layer with a thickness of about 5–7 μm. The contrast is significantly different from that inside the powder under light. In order to clearly observe the growth state of the nitride shells, we chose a cross section of a crushed Ti nitride powder for observation. Figure 6 presents the cross-sectional morphology and XRD pattern of the nitrided Ti powder. After nitriding treatment, the surface of titanium powder underwent a nitriding reaction to produce Ti-N compounds (TiN and Ti_2_N). Figure 6c is the EDS scanning characterization of the powder. It can be clearly seen that the content of N element increased significantly in the shell of Ti powder, indicating that the nitride shell was successfully formed on the surface of Ti powder by the nitriding process.

**Figure 7:**
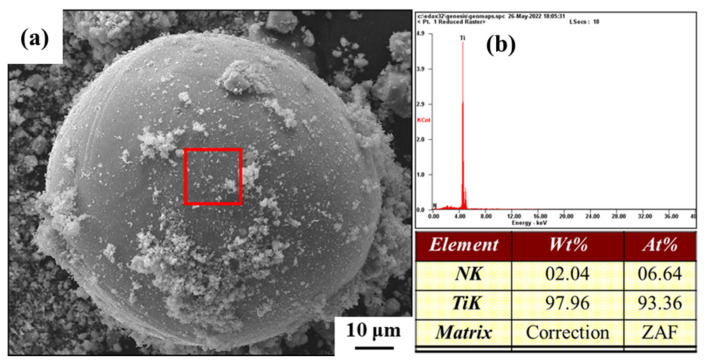
Morphology of core-shell structured mixed powders and corresponding EDS patterns. (**b**) is the results of the elements characterization in the red boxed area of (**a**).

As a comparison, we prepared a control group of TiN particles coated with Ti powder by mechanical ball milling. TiN was dispersed on the surface of Ti powder by ball milling, and the morphology of the prepared control sample powder is shown in Figure 7. TiN powder was uniformly coated on the surface of Ti powder. 

The reinforcing phase is more uniformly distributed on the surface of Ti-cores in the mixed powder obtained by nitriding treatment compared with ball milling (as demonstrated in Figure 7), which is conducive to obtaining core-shell structured composites with a more uniform microstructure. The ball milling process used in this paper did not affect the shape of the raw Ti powder, and after ball milling, the mixed powder still exhibited a spherical shape.

### 3.2. Microstructure of Ti-Based Composites with a Network-TiN

**Figure 8:**
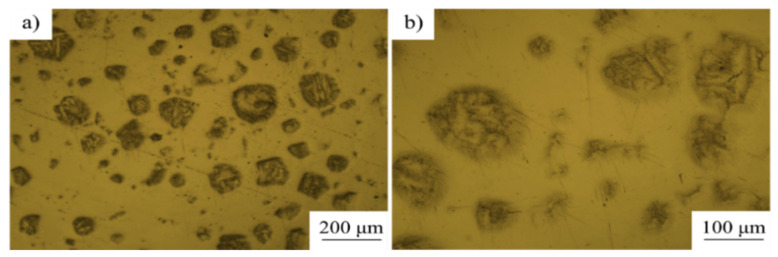
Microstructure of the Ti-N/Ti composite by in-situ synthesis. (**b**) is an amplification of (**a**).

**Figure 9:**
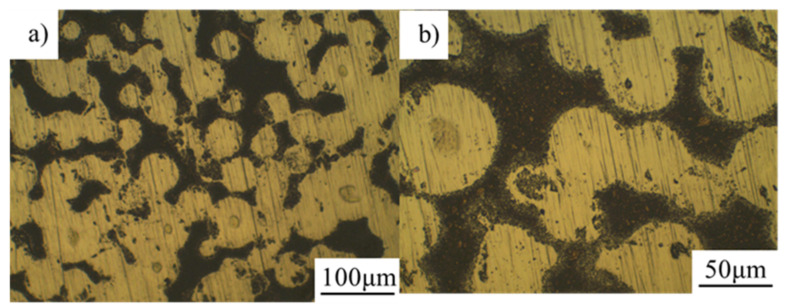
Microstructure of TiN/Ti composite by ball-milling added. (**b**) is an amplification of (**a**).

The microstructure of TiN/Ti composites prepared by in-situ synthesis and ball milling is shown in Figure 8 and Figure 9.

Figure 8 showed that there were no obvious voids in the composites, and the bonding between Ti powder and shell was relatively close. Combined with Figure 4, it could be seen that the Ti powder was black and the TiN shell was a light color in the metallographic field. It could be seen from Figure 8 that the TiN shell was sintered into a continuous region during the SPS process, which realized the initial design of the network composite structure. 

The control group prepared by directly dispersing TiN by ball milling is shown in Figure 9. It could be seen that after ball milling, the TiN powder was unevenly distributed on the surface of the spherical Ti powder in the form of clusters. However, due to the uneven dispersion of ball milling, some Ti powders were sintered together to form large particles, and no connected network structure was formed. At the same time, TiN had serious agglomeration, and there was no sintered structure between TiN.

### 3.3. The Effect of Network TiN on the Mechanical Properties of Composites

#### 3.3.1. Mechanical Property

**Figure 10:**
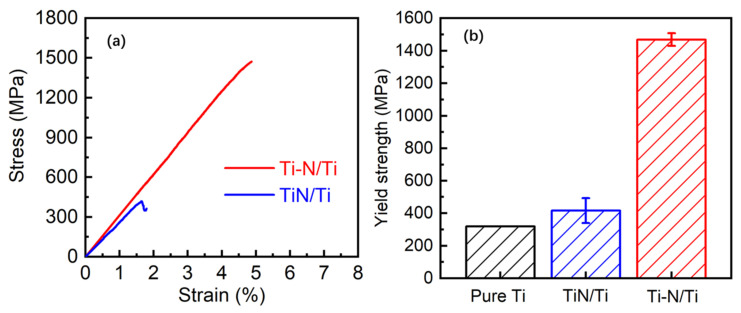
The compressive mechanical properties of the composites. Network Ti-N/Ti composites were prepared using nitrided Ti powder. TiN/Ti was the control prepared using the mechanical ball milling process. (**a**) strain-stress curves; (**b**) yield strength (the yield strength of pure Ti is obtained from Ref. [34]).

The compression performance test results of the composites are shown in Figure 10. Figure 10a demonstrates the compressive stress–strain curves of the core-shell structure composites TiN/Ti and Ti-N/Ti. It can be seen that the plastic deformation phase is not obvious in the stress–strain curves of the composites, which proves that the introduction of a large number of brittle reinforcements significantly increases the brittleness of the composites. The strength and plasticity of TiN/Ti composites prepared by ball milling are very low, the compressive strength is only 415.2 MPa, and the elongation is only 1.6%. This is because TiN agglomeration leads to stress concentration and brittle fracture of the composite. The reinforcement of Ti-N/Ti is generated by the in-situ reaction of Ti and nitrogen. The high interfacial bonding strength and high densities result in composites with a good compressive strength of 1468.5 MPa. 

A comparison of the yield strength of the two composites is shown in Figure 10b. It can be seen that when the distribution configuration of TiN changes from agglomerate distribution to connected structure distribution, the yield strength increases from 415.2 MPa to 1468.5 MPa, which is an increase of 253%, while the elongation increases from 1.6% to 5.0%. Strong plasticity enhancement was realized. This strengthening is mainly attributed to the change of TiN distribution conformation due to the change of material preparation process. When the TiN forms a reticular connectivity structure, its stress distribution and loading are more uniform, and the TiN transmits the load more efficiently, resulting in a substantial increase in the composites’ strength and plasticity.

In comparison, the properties of the composites prepared by the nitriding method are significantly better than those of the composites prepared by the direct ball milling method. It can be seen from the microstructure characterization that in the Ti-N/Ti composites prepared by the nitriding method, the nitrides are uniformly wrapped on the surface of the Ti powder and form a three-dimensional connected structure. The TiN/Ti composites prepared by ball milling are not uniform. According to the research results of Zhang et al. [14,33], the interpenetrating structure formed by the network connection improves the interface shear stress and greatly improves the load transfer strengthening. At the same time, the reinforcement plays a bridging role after the crack propagation, so that the plasticity of the material also increases greatly. Therefore, the composite material exhibits the characteristics of both strong plasticity and enhancement.

**Figure 11:**
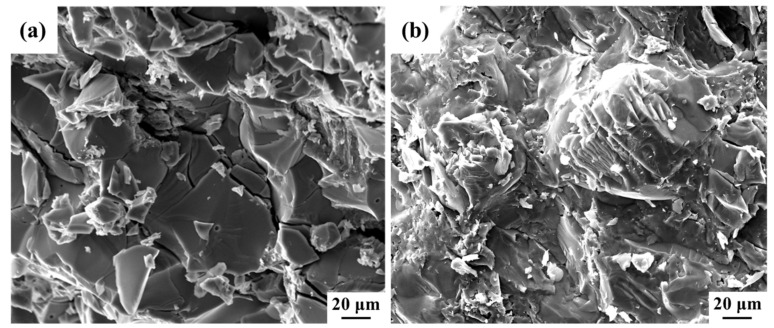
Fractured surface morphology of the composites. (**a**) TiN/Ti prepared by the ball milling process. (**b**) Network Ti-N/Ti composites prepared by nitrided Ti powder.

Figure 11 shows the room-temperature compression fracture morphology of TiN/Ti and Ti-N/Ti network structure composites. It can be seen that the fracture of TiN/Ti composites is flush, showing an obvious river pattern, and small step-like planes can be observed locally, which is consistent with the brittle fracture characteristics of the composites in the compression curves. In contrast, the fractures surface of Ti-N/Ti composites are uneven, as shown in Figure 11b, which suggests that the crack expansion path in the composite is increased, causing the composite to absorb more energy before fracture. This explains why both Ti-N/Ti composites have better strength compared to the TiN/Ti composite.

#### 3.3.2. Hardness

Hardness reflects the ability of a material to resist deformation under pressure and to produce permanent damage. In addition, hardness also reflects to a certain extent the strength and wear resistance of the material. For composite materials, hardness is determined by both the matrix and the reinforcement, which includes the physical properties of the matrix and reinforcement materials themselves as well as the distribution of the reinforcement. 

**Figure 12:**
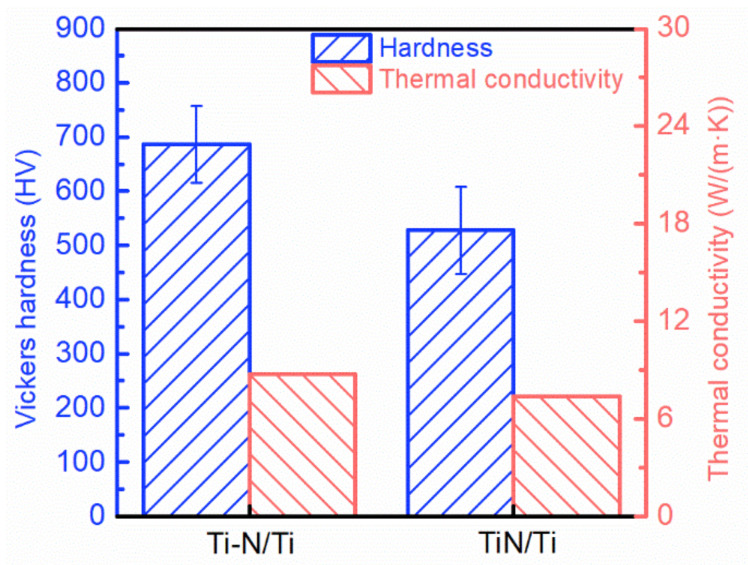
Vickers hardness and thermal conductivity of composites with a core-shell structure.

The hardness of the Ti-based composite is displayed in Figure 12. Among the three composites, the TiN/Ti composite has a lower hardness of 528.1 HV. Combined with the microstructure images in Figure 9, it can be seen that the reinforcement cannot exhibit a good strengthening effect since the complete connect structure is lacking in the composite, and therefore the hardness is low. When the Ti-N reinforcement forms a complete network structure in the composite, the hardness of the composite increases significantly, reaching 685.7 HV.

#### 3.3.3. Thermal Conductivity

The thermal conductivity of composites depends on: (1) the thermal conductivity of the reinforcement and the matrix itself; (2) the interfacial thermal resistance in the composite. For core-shell structured titanium matrix composites, the shell layer composed of the ceramic phase is continuously distributed in the matrix, which provides a path for the transfer of phonons in the thermal conductivity process, but the material defects and abundant interfaces generated by sintering will impede the thermal conductivity of the composites.

Figure 12 displays the thermal conductivity of the two composites. The TiN/Ti system had the lower thermal conductivity of 7.37 W/(m·K). It can be inferred based on the results that the thermal conductivity of the core-shell structured Ti-based composites is influenced by the structural integrity; the more complete and uniform the core-shell structure is, the higher the thermal conductivity of the composites. The interconnected structure of Ti-N/Ti composites prepared by the nitriding method causes them to have more outstanding thermal properties 8.77 W/(m·K).

## 4. Conclusions

In this paper, Ti-N/Ti network structure composites were prepared by the nitriding method. The unconnected TiN/Ti composites prepared by ball milling were used as a comparison. It can be seen from the microstructure characterization that the TiN/Ti composites prepared by ball milling show the characteristics of TiN distribution on the surface of Ti powder, but TiN does not form a connected structure after sintering. In the Ti-N/Ti composites prepared by the nitriding method, the TiN shell was completely formed on the surface of Ti powder, and the core-shell structure was prepared. Compared with the TiN dispersion achieved by using mechanical ball milling, the Ti-N shells were prepared on the surface of Ti powder by the nitriding method, and their distribution was more uniform than that of mechanical ball milling, and the Ti-N shells were in contact with each other and connected during the SPS sintering process, forming a network structure. The hardness of the Ti-N/Ti core-shell composite is 685.7 HV and the compressive strength is 1468.5 MPa. The high mechanical properties are mainly attributed to the effective load of the connected shell structure.

## Data Availability

The data presented in this study are available on request from the corresponding author.
